# A Mechanism of Male Germ Cell Apoptosis Induced by Bisphenol-A and Nonylphenol Involving ADAM17 and p38 MAPK Activation

**DOI:** 10.1371/journal.pone.0113793

**Published:** 2014-12-04

**Authors:** Paulina Urriola-Muñoz, Raúl Lagos-Cabré, Ricardo D. Moreno

**Affiliations:** Departamento de Fisiología, Facultad de Ciencias Biológicas, Pontificia Universidad Católica de Chile, Santiago, Chile; Southern Medical University, China

## Abstract

Germ cell apoptosis regulation is pivotal in order to maintain proper daily sperm production. Several reports have shown that endocrine disruptors such as Bisphenol-A (BPA) and Nonylphenol (NP) induce germ cell apoptosis along with a decrease in sperm production. Given their ubiquitous distribution in plastic products used by humans it is important to clarify their mechanism of action. TACE/ADAM17 is a widely distributed extracellular metalloprotease and participates in the physiological apoptosis of germ cells during spermatogenesis. The aims of this work were: 1) to determine whether BPA and NP induce ADAM17 activation; and 2) to study whether ADAM17 and/or ADAM10 are involved in germ cell apoptosis induced by BPA and NP in the pubertal rat testis. A single dose of BPA or NP (50 mg/kg) induces germ cell apoptosis in 21-day-old male rats, which was prevented by a pharmacological inhibitor of ADAM17, but not by an inhibitor of ADAM10. *In vitro*, we showed that BPA and NP, at similar concentrations to those found in human samples, induce the shedding of exogenous and endogenous (TNF-α) ADAM17 substrates in primary rat *Sertoli* cell cultures and TM4 cell line. In addition, pharmacological inhibitors of metalloproteases and genetic silencing of ADAM17 prevent the shedding induced *in vitro* by BPA and NP. Finally, we showed that *in vivo* BPA and NP induced early activation (phosphorylation) of p38 MAPK and translocation of ADAM17 to the cell surface. Interestingly, the inhibition of p38 MAPK prevents germ cell apoptosis and translocation of ADAM17 to the cell surface. These results show for the first time that xenoestrogens can induce activation of ADAM17 at concentrations similar to those found in human samples, suggesting a mechanism by which they could imbalance para/juxtacrine cell-to-cell-communication and induce germ cell apoptosis.

## Introduction

Apoptosis is a regulated form of cell death and plays an important role in the events leading to germ cell differentiation during mammalian spermatogenesis. Several intrinsic and extrinsic factors induce an up-regulation of apoptosis, which leads to decreased sperm production that has been related to human male infertility [1]–[3].

It is believed that the function of apoptosis during spermatogenesis is to balance the number of germ cells to Sertoli cells in order sustain proper proliferation and differentiation during spermatogenesis. We have previously shown that the induction of germ cell apoptosis in rats can be regulated by activation of the transmembrane metalloprotease ADAM17 (A-Disintegrin and Metalloprotease-17) [4]–[6]. ADAM17 belongs to a family of metalloproteases that are structurally consisted of an N-terminal signal peptide, followed by a prodomain, a metalloprotease domain, a disintegrin domain, a cysteine-rich region, an EGF-like domain, a transmembrane region and a cytoplasmic domain. Depending of their tissue expression pattern and function, some of the ADAM members may lack the metalloprotease domain (e.g. ADAM1) or have specific point mutations that render them inactive [7]. In the case of ADAM17, it is involved in the shedding of many protein ectodomains from the cell surface, including TNF-α, c-kit, FasL, Notch, APP and TrkA, among others, indicating strong participation in autocrine, paracrine and juxta/paracrine signaling [8], [9]. One of the most interesting topics in ADAM protein biology is their regulation in different cellular contexts. Most models have shown basal (constitutive) and inducible shedding activity in different cell types [18]. In this sense, it has been reported that ADAM17 shedding activity may be regulated by p38 MAPK kinase and by phorbol ester (PMA), suggesting the involvement of protein kinase C (PKC) [10], [11]. Some reports have shown that phosphorylation of the intracellular domain at Thr735 by p38MAKP and trafficking to the cell surface are important steps in the shedding of substrates like TGF-α and TNF-α [12], [13]. In addition, it seems that ancillary proteins such as Annexins, CD9 and irhom1/2 regulate the activity and substrate selectivity of ADAM17 [14]–[16].

We have previously shown that meiotic germ cells (spermatocytes) undergoing apoptosis harbor an active form (phosphorylated) of ADAM17 that is localized at the cell surface, and that these cells also lack the extracellular domain of c-kit [6], suggesting that the shedding of the c-kit extracellular domain by ADAM17 could in some way induce apoptosis. In addition, PMA stimulate *in vivo* germ cell apoptosis and induce fragmentation of the extracellular domains of c-kit. Physiological and PMA-induced germ cell apoptosis could be prevented by using GW280264X, a pharmacological inhibitor of ADAM17 [6]. On the other hand, treatment with etoposide, which induces DNA fragmentation, promotes germ cell apoptosis, and up-regulation of ADAM17 protein and mRNA levels *in vivo* and *in vitro* [5], [17], [18]. In addition, etoposide-induced germ cell apoptosis could be prevented by using pharmacological inhibitors of ADAM17 and the related isoform ADAM10 [5], [17]. Interestingly, heat stress, which also induces germ cell apoptosis, does not induce activation of ADAM17 or ADAM10, suggesting that these enzymes are selectively activated by specific stimuli.

In recent years, several countries have experienced increases in the incidence of cryptorchisms and hypospadias [19], [20], which are the most frequent congenital malformations in young boys, along with a trend in sperm count decline. It has been proposed that modern lifestyle and daily exposure to environmental toxicants (endocrine disruptors) could promote these reproductive disorders [21].

Endocrine disruptor chemical (EDC) is the common name given to a wide variety of molecules that are capable of inducing estrogenic and/or anti-androgenic responses in adult and infant animals, including humans. In industry, Bisphenol A [2,2-bis(4-hydroxyphenyl)propane] (BPA) is used to harden polycarbonate plastics in a wide variety of products, such as baby bottles, lunch boxes, toys and water pipes [22]. On the other hand, alkylphenolic compounds, such as 4-nonylphenol (NP), and their polyethoxylates are used as nonionic surfactants for the enhancement of products, or in processes where foaming, emulsification, solubilization, or dispersion are important, such as in the production of pesticides and paints. BPA and NP behave like EDC and have been detected in human samples, including serum, urine, amniotic fluid of pregnant women, breast milk and semen [23]–[30], suggesting a potential risk in the development of human genital malformations and reproductive problems. Interestingly it has been showed that NP and BPA induce *in vivo* germ cell apoptosis in male rats, suggesting that both compounds could have similar targets in the testis [31], [32]. In the same regard, the exposure of male rats to the toxicant Mono-(2-ethylhexyl)phthalate (MEHP), which induces germ cell apoptosis, results in the release of soluble TNF-α from germ cells, which leads to a robust induction of FASL by Sertoli cells, and, in turn, may induce apoptosis in germ cells. It has been reported that matrix-metalloproteinase 2 (MMP2) could be involved in the release of TNF-α in the rat testis after MEHP treatment [33]. However, the same authors also observed an early increase in protein levels of ADAM17 and ADAM10, suggesting that these metalloproteases could also participate in germ cell apoptosis induced by toxicants in the mammalian testis. Therefore, it is not difficult to hypothesize that BPA and NP induce the activation of ADAM17, which leads to germ cell apoptosis in male rats. The aims of this work were: 1) to determine whether BPA and NP induce ADAM17 activation; and 2) to study whether ADAM17 and/or ADAM10 are involved in germ cell apoptosis induced by BPA and NP in the pubertal rat testis.

## Materials and Methods

### Animals

Male Sprague–Dawley rats of 17- and 20-days old were acquired from the Animal Facility in Faculty of Biological Science, Pontifical Catholic University of Chile. The rats were housed under a 12L∶12D cycle with water and rat chow *ad libitum* and were euthanized by cervical dislocation.

### Ethical statement

All the experiments in this work were conducted in accordance with the rules laid down by the Consortium for Developing a Guide for the Care and Use of Agricultural Animals in Agricultural Research and Teaching and by the National Research Council. All experimental protocols perform in this work were reviewed and approved by the Chilean National Fund of Science and Technology (FONDECYT), and the “Bioethical and Biosecurity Committee” of the Faculty of Biological Sciences (permit number CBB-167/2010). Ricardo Moreno served on the Bioethics and Biosafety Committee at Pontificia Catholic University during the time when the animal research protocol was submitted for review and approval. However, he recused himself from the review and approval of his research group's protocol.

### Chemicals and antibodies

Bisphenol A [2,2-bis(4-hydroxyphenyl)propane] (BPA) and 4-nonylphenol (NP) were obtained from Sigma (St Louis, MO, USA). The p38 MAPK inhibitor, PD169316 (513030), and a general metalloprotease inhibitor, BB-94 (Batimastat), were obtained from Calbiochem (San Diego, CA, USA). Inhibitors GI254023X and GW280264X were kindly donated by Dr. Andreas Ludwig (Christian-Albrechts-University, Kiel, Germany). LipofectAMINE 2000 and p-nitrophenyl phosphate (p-NNP), the substrate of alkaline phosphatase, were obtained from Invitrogen (Carlsbad, CA, USA). TUNEL assay, Reverse Transcriptase and PCR Master Mix were obtained from Promega (Madison, WI, USA). TRIzol-Reagent, rat TNF-α ELISA kit and Propidium Iodide (P1304MP) were obtained from Invitrogen (Carlsbad, CA, USA).

Rabbit polyclonal antibody against ADAM17 (ab39163), which reacts with an epitope located in the activation site (cysteine switch and furin cleavage site) of ADAM17 [34]–[36], and p38 (phospho Y182+T 180) were purchased from Abcam (Cambridge, MA, USA), Rabbit polyclonal antibody against PARP-1/2 (sc-7150), clusterin-α (sc-8354), total-p38 MAPK (sc-7149) and mouse monoclonal SCP-3 (sc-74569) were purchased from Santa Cruz Biotechnology (Santa Cruz, CA, USA). Rabbit polyclonal antibody against Cleaved Caspase-3 (Asp175) (#9661) was purchased from Cell Signaling (Danvers, MA, USA). Mouse monoclonal antibody against β-actin (AC-15) and anti-rabbit IgG-FITC (F0382) were purchased from Sigma (St Louis, MO, USA). Peroxidase anti-mouse IgG and peroxidase anti-rabbit IgG were obtained from KPL (Gaithersburg, MD, USA). Anti-rabbit UltraVision Detection Systems, EZ-Link Sulfo-NHS-SS-Biotin and NeutrAvidin Agarose Resins were obtained from Thermo Scientific (Fremont, CA, USA). Western Lightning Chemiluminescense Reagent Plus kit was obtained from PerkinElmer Inc. (Waltham, MA, USA).

### Experimental procedure

Individual male rats (20-day-old) were weighed and randomly located to experimental or control (vehicle) groups. After treatment all animals were left in indivual cages under a 12L∶12D cycle with water and rat chow ad libitum. Usually this procedure was performed between 9–10 A.M. and involved the treatment of experimental and vehicle animals in parallel. Even after 24 h of treate no animal showed any sign of distress, infection of any anomalous behavior. This experimental protocol was design to evaluate *in vivo* the effect of BPA and NP upon germ cell apoptosis and the role of ADAM17 in this process.

### Xenoestrogens injections

Individual male rats (20-day-old) were weighed and then intraperitoneally injected (ip) with a single dose of BPA or NP (stock solutions were dissolved in ethanol) dissolved in olive oil. The injected volume never exceeded 20 µl. Rats were left for different periods of time under the same conditions described above and then sacrificed by cervical dislocation.

### Intra-testicular injections

Twenty-day-old male rats were anesthetized with an intra-muscular (i.m.) injection of a mixture of ketamine∶xylazine (1 mg/kg∶750 mg/kg). The testes were exteriorized through a low midline incision. Ten microliters of a solution containing 10 µM of GW280264X, GI254023X and or 5 µM of PD 169316 was infused via a 30G needle inserted through the tunica albuginae with the tip resting in the testicular interstitium [6], [37]. Following drug delivery, the testes were returned to the peritoneum and the incision was closed. As a control, ethanol (at the same dilution than drugs) diluted in sterile PBS was injected into the testes. To ameliorate surgical pain, 5 mg/kg Ketoprofen was administrated prior to recovery. Water and food ingestion was monitored following surgery until the end of the experiment (up to 24 h). At least three different rats were used for each experiment (n = 3).

### Protein extraction and Western blotting

Protein extraction was performed by homogenizing isolated decapsulated testes or isolated testicular cells in a buffer containing 1 M NaCl, 1 mM EDTA, 10 mg/ml PMSF, 1% Triton X-100, 20 mM Tris–HCl pH 7.4, plus a protease inhibitor cocktail (Sigma, St Louis, MO, USA) including 2 mM AEBSF [4-(2-Aminoethyl) benzenesulfonylfluoride hydrochloride], 0.3 µM aprotinin, 130 µM bestatin hydrochloride, 14 µME-64, 1 mM EDTA, and 1 µM leupeptin hemisulfatein, and then centrifuging for 10 min at 10,000× g at 4°C. The samples were run on a 10% polyacrylamide gel (SDS–PAGE) under reducing and denaturing conditions, and then transferred to nitrocellulose at 400 mA for 2 h. Nitrocellulose was blocked with 3% (w/v) non-fat milk, 0.1% Tween in PBS or TBS, pH 7.4, and then incubated overnight at 4°C with one of the following antibodies: anti-p38 MAPK (pThr180/Tyr182) phosphospecific (0.125 mg/ml), anti-total p38 MAPK, anti-ADAM17, anti-SCP-3, anti-clusterin (0.2 mg/ml) or anti-β-actin (0.3 mg/ml), as a loading control. Membranes were then incubated with a secondary antibody conjugated with horseradish peroxidase-secondary antibodies (KPL, Gaithersburg, MD) diluted 1∶3,000 in blocking solution for 1 h at room temperature. Protein bands were revealed using the electrochemiluminescence kit (PerkinElmer Inc., Waltham, USA).

### Testes histology

Rat testes were fixed in Bouin solution (picric acid-aqueous solution∶formaldehyde∶glacial acetic acid, 15∶5∶1). Tissues were fixed at least overnight at room temperature. Then, they were embedded in paraffin, sectioned into 5 µm thick slices and mounted on xylanized slides. Histological sections were deparaffinized through a xylol series, hydrated through an alcohol series and then washed with water. Slides were stained with periodic acid-Schiff (PAS) and hematoxylin, according to standard procedures. Slides were observed under an Olympus BH-2 microscope (Olympus, Tokyo, Japan). Images were acquired by a digital Nikon photo camera model CoolPix 4500 (Nikon, Tokyo, Japan). The apoptotic index was calculated as the average number of apoptotic (pyknotic) cells per seminiferous tubule cross-section. Three testicular histological sections per rat were used (n = 3, a total of nine sections), and 100 randomly selected tubules was counted in each tissue section (a total of 900 tubules were recorded per treatment).The data represent the mean (SD) ± SEM.

### Immunohistochemistry

Active caspase-3 was detected in paraffin-embedded cross-sections of rat testes fixed in Bouin's solution and treated with sodium citrate 0.01 M, pH 6, to expose the antigens. The samples were first treated with 3% H_2_O_2_ for 10 min, then, to prevent unspecific binding, a standard protein block system (Ultra V block, Thermo Scientific, Fremont, CA) was applied for 10 min. Primary antibody, which detects p38 MAPK only when two residues (threonine-180 and tyrosine-182) are phosphorylated, was applied at a concentration of 2 µg/ml; samples were incubated overnight at 4°C in a humidified chamber after being washed three times for 5 min in a Tris–HCl buffer, pH 7.6, with 0.3 M NaCl and 0.1% Tween-20. Biotinylated secondary antibody, streptavidin–biotinylated–peroxidase complex, amplification reagent (biotinyl tyramide) and peroxidase-conjugated streptavidin were applied step-by-step for 10 min each (Thermo Scientific, Fremont, CA). Afterwards, slides were washed three times for 5 min in a Tris–HCl buffer, pH 7.6, with 0.3 M NaCl and 0.1% Tween-20. Finally, DAB (3,3-diaminobenzidine tetrahydrochloride) Plus Substrate and DAB Plus Chromogen (Thermo Scientific, Fremont, CA) were applied for 1 min and washed in distilled water. Samples were stained with hematoxylin and observed under a phase contrast microscope (Optiphot-2, Nikon, Tokyo, Japan) and photographed with a digital camera (CoolPix 4500, Nikon, Tokyo, Japan).

### Sertoli cells isolation and culture

Sertoli cells were obtained from the testes of 17-day-old male Sprague-Dawley rats that had been killed by cervical dislocation. Testes were removed, decapsulated and placed in PBS containing 0.1 mg/ml collagenase (Sigma, St Louis, MO). Then, the tubules were washed three times in PBS. Tubule cell isolation was performed by mechanical disaggregation in the presence of 0.1 mg/ml DNase (Sigma, St Louis, MO) using a 21G needle from different segments of the seminiferous tubules that were previously isolated in PBS. Then, the solutions were filtered through a mesh with a pore diameter of 200 µm and another with a pore diameter of 70 µm. Cells were resuspended in a solution containing PBS and distilled water (1∶9) to produce hypotonic shock, which destroys germ cells but not Sertoli cells. Then, the cells were filtered again, and the filtered solutions were centrifuged for 3 min at 800× g and resuspended in DMEM-F12 medium without serum, and containing only 10% antibiotic and anti-mycotic (Gibco, Invitrogen, Carlsbad, CA). Following this, 1×10^6^ cells were cultured in DMEM-F12 medium supplemented with 10% antibiotic and anti-mycotic, pH 7.2, at 37°C and in 5% CO_2_. After 24 h, the cells were washed and cultivated with fresh medium, in the same conditions as above for 4 days, after which, every germ cell had been phagocytosed by Sertoli cells. The culture obtained had at least 90% purity; the other 10% was composed of peritubular cells.

### Immunofluorescence

The localization of ADAM17 was assayed in isolated Sertoli cells fixed in 4% paraformaldehyde in PBS (pH 7.4) for 15 min and then permeabilized in cold methanol for 5 min. Then, the cells were placed on a slice with 0.1% polylysine. To prevent unspecific binding, the samples were treated with 3% BSA in TBS containing 0.1% Tween-20 for 1 h at room temperature. Primary antibody against ADAM17, diluted in 3% BSA-TBS containing 0.1% Tween-20, was applied to the slice at a concentration of 1 µg/ml, before being incubated overnight at 4°C in a humidified chamber, after the slice had been washed three times for 10 min in TBS containing 0.1% Tween-20. Then, anti-rabbit FITC diluted 1∶100 in 3% BSA in TBS containing 0.1% Tween-20 was applied to the slice and samples were incubated for 1 h at room temperature, after the slices had been washed three times for 10 min in TBS containing 0.1% Tween-20. Next, propidium iodide (100 µM) diluted 1∶5,000 in distillated water was applied to the slice and samples were incubated for 5 min at room temperature, before being washed as above. Finally, the slices were mounted with fluoromount (Sigma, St Louis, MO) and observed under a microscope (Zeiss LSM-510, Germany).

### TUNEL analysis

Apoptotic fragmentation of DNA was evaluated by TUNEL analysis in deparaffinized sections of rat testes. The DeadEnd^TM^ Fluorometric TUNEL System Kit was used according to the manufacturer's instructions (Promega, Madison, WI, USA). Samples were observed under phase contrast (Nikon model Optiphot-2, Tokyo, Japan) and micrographs were taken with a digital camera (Nikon model CoolPix 4500, Tokyo, Japan). TUNEL-positive germ cells, visualized as green cells, were quantified in each tissue section by counting the number of TUNEL-positive cells per seminiferous tubule cross-section in random areas. Three testicular histological sections were taken per rat (three rats total, n = 3), and 100 randomly selected tubules were counted in each tissue section (a total of 900 tubules per treatment). The data represent the mean (SD) ± SEM.

### Surface ADAM17 detection by flow cytometry

Isolated intra-tubular cells (i.e. germ and Sertoli cells) were obtained from 21-day-old rats previously treated with BPA or NP (50 mg/kg), with or without inhibitors GW280264X, GI254023X and/or PD169316. Cells were blocked with DMEM-F12 supplemented with 3% BSA for 1 hour. Anti-ADAM17 antibody was diluted 1∶100 in the same blocking buffer and incubated at 4°C overnight. Then, cells were washed 3 times in PBS and incubated with anti-rabbit secondary antibody FITC conjugated for 1 hour. Next, cells were washed three times with PBS and the final pellet dissolved in PBS. The samples were analyzed by a flow cytometer (FAScanto) and 10,000 gated events were acquired in each sample. One sample was used as autofluorescence, while another was incubated with only primary antibody and the third sample with only secondary antibody. All data were analyzed using the FCS express V4.0 software (De Novo Software, Los Angeles).

### Cell cycle analysis by flow cytometry

The testes from rats treated with different concentrations of BPA for 24 h were removed, decapsulated and placed in PBS containing 0.1 mg/ml collagenase (Sigma, St Louis, MO). Then, the tubules were washed three times in PBS. Tubule cell isolation was performed by mechanical disaggregation in the presence of 0.1 mg/ml DNase (Sigma, St Louis, MO) using a 21G needle from different segments of the seminiferous tubules that were previously isolated in PBS. Then, the solutions were filtered by through a mesh with a pore diameter of 200 µm and another with a pore diameter of 70 µm. To analyze cell cycles, the individual cells were fixed in 70% ethanol overnight. The cells were pelleted and washed once with PBS. Then, the pellet was then dissolved in a cell cycle buffer containing 0.1% sodium citrate, 0.3% Triton X-100 (Sigma, St. Louis, MO, USA), 50 mg/ml propidium iodide and 50 mg/ml RNase A (Invitrogen, Carlsbad, CA, USA) dissolved in distilled water. The samples were analyzed within 10 min of buffer addition on a flow cytometer (FAScanto) and 10,000 gated events were acquired for each sample. All data were analyzed with the software FCS express V4.0 (De Novo Software, Los Angeles).

### Quantification of soluble TNF-α

Soluble TNF-α was measured using the *TNF-α Rat ELISA Kit* (Invitrogen, Carlsbad, CA, USA). The lowest concentration detected by this assay is 4 pg/ml of rat TNF-α. Briefly, primary culture Sertoli cells were treated with 10 µM BPA or NP with or without 10 µM BB-94 for 6 hours, before the supernatant was collected and centrifuged at 1,000× g for 3 min to eliminate cellular debris. The culture medium was diluted in a standard buffer (included in the kit) and incubated in a 96-well plate coated with antibody against rat TNF-α. Then, the sample was washed and a secondary antibody coupled to biotin was added to the wells and incubated for 90 min at room temperature. Finally, the mixture was incubated with streptavidin coupled to peroxidase for 30 min at room temperature. The reaction was measured at 450 nm in an ELISA reader ELx800^TM^ (BioTek, USA).

### TM4 cell line transfection

TM4 cell lines were transfected with the plasmid (AP)-NRG β1, which was kindly donated by Dr. Carl Blobel (Hospital for Special Surgery, New York, USA), or the mouse ADAM17shRNA (pG4-T shRNA ADAM17 y pG4-T scramble), which was kindly donated by Dr. Yan (Tongji Medical College, Wuhan, China) [38]. Briefly, TM4 cells at 80% confluence cultivated for 12 h in DMEM-F12 medium with 10% FBS and 10% antibiotic and anti-mycotic. Then, the cells were washed and cultured in DMEM-F12 medium deprived of serum, antibiotic and anti-mycotic for 1 h. Later, cells were cultured for 6 h with a complex DNA-LipofectAMINE 2000, and cultured for another 24 or 48 h in DMEM-F12 medium with 10% FBS and 10% antibiotic and anti-mycotic. Transfection was evaluated by the presence of neuregulin α1 or the absence of ADAM17 by RT-PCR.

### Alkaline Phosphatase activity measurement

Alkaline Phosphatase activity was measured from the culture medium of TM4 cells treated with BPA or NP. The medium was collected and incubated for 1 h with p-NPP (p-nitrophenyl phosphate) (4∶1), which is a colorimetric and soluble alkaline phosphatase substrate, and then measured in a spectrophotometer at 450 nm (ELx800^TM^, BioTek, USA).

### RNA extraction and RT-PCR

Total RNA of TM4 cells was isolated using TRIzol-Reagent (Invitrogen, Carlsbad, CA) according to the manufacturer's recommendations. Total RNA was quantified, and after confirmation of its integrity, cDNA was generated from 1 µg RNA using random primers and Reverse Transcriptase (Promega, Madison, WI). The cDNA obtained was amplified by polymerase chain reaction (PCR) in 30 cycles using the PCR Master Mix (Promega, Madison, WI). Several primer sets were used to obtain the PCR products: ADAM17 forward 5′-GTTGGTGAGCCTGACTCTA-3′ and reverse 5′-CCTCTTGTGGAGACTTGA-3′; Neuregulin β1 forward 5′-ATCCACGACTGGGACCAG-3′ and reverse 5′-AAGCTTCTGCCGCTGTTTC-3′; and GAPDH forward 5′-TCCACCACCCTGTTGCTGTA-3′ and reverse 5′-ACCACAGTCCATGCCATCAC-3′; these were run using the same conditions as previously described [18]. Aliquots of the PCR products were run on a 1% agarose gel and then stained with SYBR Green (Invitrogen, Carlsbad, CA, USA). Bands obtained were analyzed measuring the pixels with AdobeR Photoshop 7.0 (Adobe System Incorporated, USA), and normalized to GAPDH mRNA levels.

### Statistical analysis

For mean comparisons, we used analysis of variance (ANOVA). When the ANOVA test showed statistical differences, the Tukey post hoc test was used to discriminate between groups. Statistical significance was defined as p<0.05. Statistical analyses were performed using GraphPad Prism version 5.0 for Windows (GraphPad Software, San Diego California USA, www.graphpad.com).

## Results

### BPA and NP induce germ cell apoptosis in pubertal male rats

**Figure 1:**
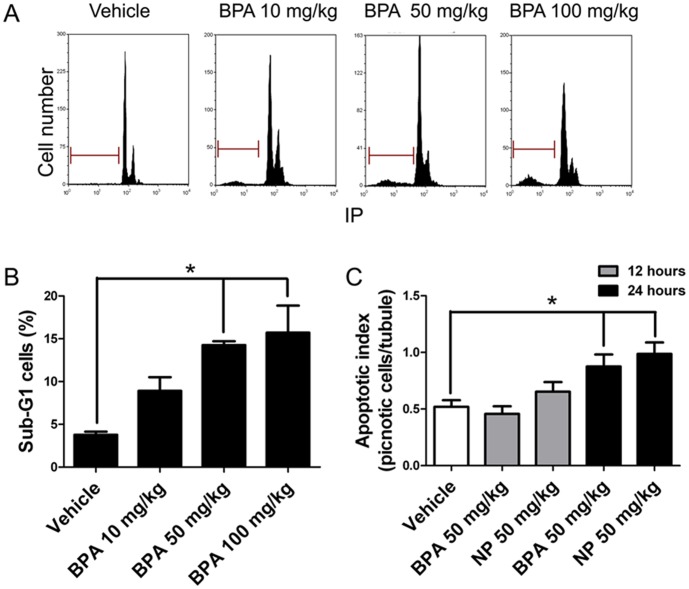
BPA and NP induce apoptosis in rat testes. A) Cell cycle analysis by flow cytometry of seminiferous tubule cells isolated from 21 day-old rats after 24 h of a single dose of 10, 50 or 100 mg/kg BPA. The horizontal bar indicates the sub-G1 cell population, which is a parameter of apoptosis. B) There is a significant increase in the percentage of sub-G1 cell population at 50 and 100 mg/kg. C) Quantification of pyknotic cells after the injection of 50 mg/kg of BPA or NP. * p<0.05, n = 3.

**Figure 2:**
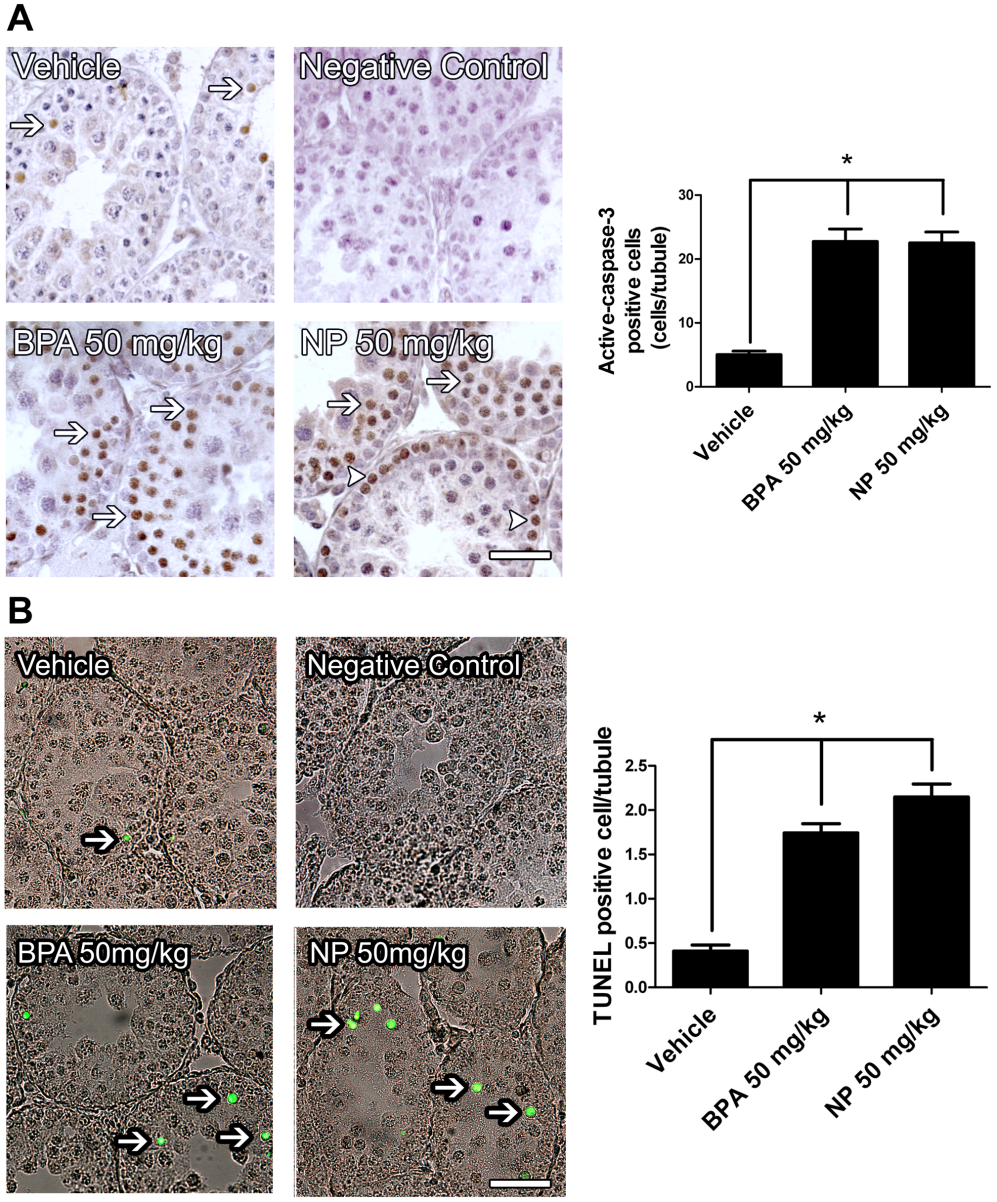
A single dose 50 mg/kg of BPA and NP induces a significant increase in active caspase-3 and TUNEL-positive cells in rat testis. A) BPA and NP treatment induce an increase in the number of active caspase-3. Note that caspase-3 positive cells were mainly spermatocytes (arrows) with BPA, whereas mainly spermatogonia were seen in the case of NP (arroheads). B) TUNEL-positive cells (arrows). * p<0.05, n = 3. Bars 50 µm.

First, we aimed to determine the doses at which BPA or NP induces apoptosis in pubertal rat testes *in vivo*. To this end, 21-day-old male rats were subcutaneously injected with different doses of BPA and germ cell death was assayed 24 h later. We chose this experimental protocol because previous investigations stated that orally administered BPA and NP are rapidly absorbed and transformed to glucuronide derivatives during passage through the gut wall, and the liver [39], [40]. In addition, this protocol has previously been successfully used for determination of the effect of EDCs in rodent models [41]. We chose to work with 21-day-old rats because it has been previously described that newborn and pubertal animals are more susceptible to EDCs than adult animals [42]. A dose-response curve using BPA showed that the percentage of sub-G1 cell population, which represents cells with fragmented and condensed DNA, increased significantly from 3.77±0.37% to 14.27±0.44% and 15.72±3.16% at 50 mg/kg and 100 mg/kg BPA, respectively (Fig. 1A and 1B). There were no differences between the percentage of sub-G1 population in 50 or 100 mg/kg treated rats. The application of 10 mg/kg BPA lead to an increase to only 8.92±1.60%, compared with the result of 3.77±0.37% in the sub-G1 population for the vehicle treatment, with no statistically significant difference. We chose 50 mg/kg as the working concentration in the subsequent experiments, since it was the smallest concentration that induced an increase in DNA fragmentation in rat testis cells *in vivo*. In addition, this dose has been previously used in similar experiments, and is within the range of the No Observed Adverse Effective Levels (NOAEL) [43]–[45]. Germ cells undergoing apoptosis are identified by counterstaining with periodic acid-Schiff and hematoxylin histological testes sections as having a round and pyknotic appearance. We have previously shown that these pyknotic cells express apoptotic markers such as active caspase-3 and stain positively for TUNEL [46]. The average number of cells with apoptotic morphology (pyknotic) within a seminiferous slide (apoptotic index) increased significantly from 0.52±0.06 to 0.88±0.10 in the case of BPA and to 0.99±0.10 in the case of NP (Fig. 1C) 24 h after treatment. There was no difference 12 h after injection. We also found that BPA or NP treatment induced an increase in active caspase-3 in germ cells but not Sertoli cells, which can be seen in histological sections (Figure 2A). Interestingly, we found that only spermatocytes (cells in meiosis) were intensely labeled in BPA-treated animals (Figure 2A, arrows), whereas spermatogonia (Figure 2A, arrowheads) and few spermatocytes were labeled in NP-treated animals. We found that the number of caspase-3-positive cells significantly increased 24 h after BPA or NP treatment (Fig. 2A). The number of active caspase-3-positive cells increased from 5.04±0.56 per seminiferous tubule in vehicle-treated testes to 22.75±1.98 in BPA- and to 22.54±1.72 in NP-treated rats (Fig. 2A). We also evaluated the fragmentation of DNA in histological sections of 21-day-old rats treated with 50 mg/Kg of BPA or NP using the TUNEL assay. The results showed that BPA- and NP-treated animals had 1.78±0.20 and 2.19±0.18 TUNEL-positive cells per seminiferous tubule, respectively, which was significantly higher than that reported for vehicle-treated animals (0.48±0.08 positive cells) (Fig. 2B). These results clearly indicate that BPA and NP induce apoptosis in rat germ cells.

### ADAM17 is involved in *in vivo* germ cell apoptosis induced by BPA and NP

**Figure 3:**
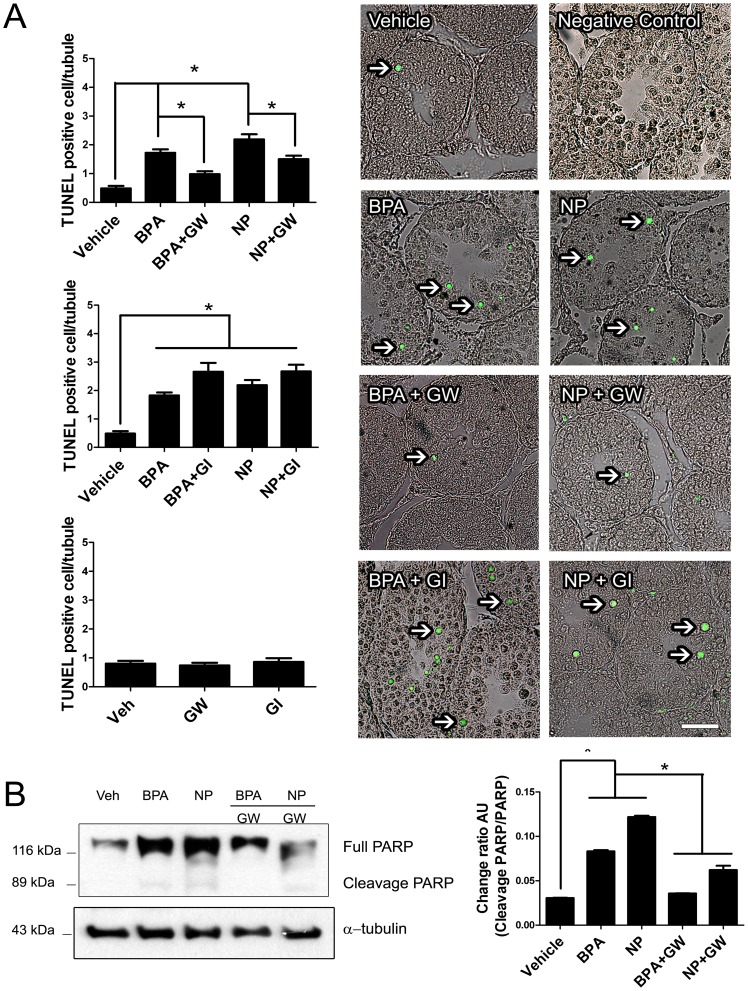
The ADAM17 inhibitor GW280264X prevents *in vivo* germ cell apoptosis induced by BPA or NP. A) Upper graph: Intra-testicular injection of 10 µM GW280264X (GW) prior prevents the increase in the number of TUNEL-positive cells in rat testes induced by 50 mg/kg BPA or NP treatment. Middle graph: Intra-testicular injection of 10 µM GI254023X (GI), an ADAM10 inhibitor, does not prevent the increase in the number of TUNEL-positive cells in rat testes induced by 50 mg/kg BPA or NP treatment. Bottom graph: The application of each inhibitor does not interfere with the normal apoptosis level. Right Panel: Representative TUNEL images from rat testis with the different treatments. B) Treatment with 50 mg/kg BPA or NP induces a significant increase in the cleaved form (89 kDa) of PARP on western blot. The addition of 10 µM GW280264X (GW) prevents the increase of the 89 kDa form in rat testes treated with BPA or NP. * p<0.05, n = 3. Bars = 50 µm.

Since we have previously found that ADAM17 and ADAM10 are involved in apoptosis induced by external compounds in the rat testis [6], [17], we decided to investigate whether these metalloproteases participate in BPA- and NP-induced apoptosis in rat testes. To this end, ADAM17 and ADAM10-inhibitors were intra-testicularly administered before the injection of 50 mg/kg BPA or NP. Results showed that the ADAM17 inhibitor, GW280264X, prevented any increase in the number of TUNEL-positive cells induced by BPA and NP, from 1.78±0.20 to 0.98±0.10 TUNEL-positive-cells in the case of BPA, and from 2.19±0.18 to 1.50±0.12 TUNEL-positive cells in the case of NP (Fig. 3A, upper panel). The values for TUNEL-positive cells in animals treated with BPA or NP plus ADAM17 inhibitor were not statistically different from those reported for controls. On the other hand, the ADAM10 inhibitor, GI254023X, did not prevent the increase in apoptosis after BPA or NP injection (Fig. 3A, middle panel). The application of GW280264X or GI254023X alone did not interfere with normal apoptosis in the rat testis (Fig. 3A, bottom panel).

As a second approach, we studied the cleavage of PARP, which is a substrate of caspase-3 that is widely used as an apoptosis marker. GW280264X prevented the cleavage of PARP in 59% and 48% of cells 24 h after the injection of BPA or NP, respectively (Fig. 3B). Given that NP and BPA induce DNA breaks, the increase in the total levels of PARP could be related to its role in sensing and initiation of DNA repair, such as been observed in other systems [47]. Thus, these results suggest that ADAM17, but not ADAM10, participates in the germ cell apoptosis induced by BPA and NP in the pubertal rat testis.

### BPA and NP induce the activation and substrate shedding of ADAM17

**Figure 4:**
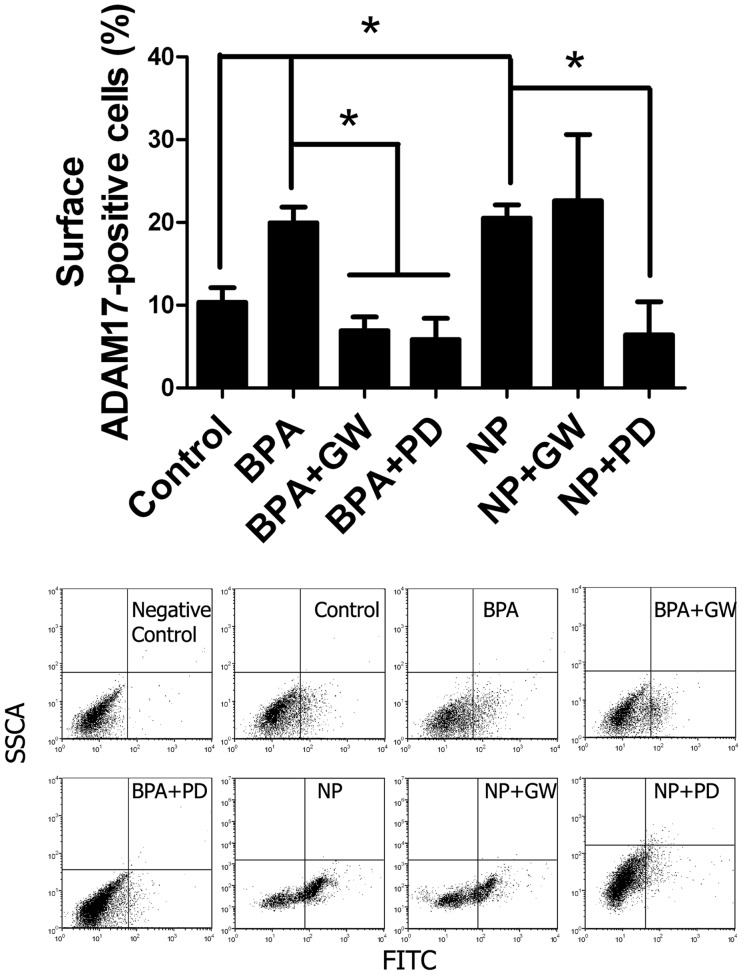
BPA and NP induce increase of ADAM17 in cell surface. A) Seminiferous tubule cells from rats injected with 50 mg/kg of BPA or NP were isolated as described in the materials and methods section and incubated with and antibody against the active form of ADAM17. The graph shows that the percentage of cells labeled with the antibody increases in rats treated with BPA or NP, but is reduced when rats were treated in the presence of GW280264X, a pharmacological inhibitor of ADAM17 (GW), or PD169316, a pharmacological inhibitor of p38 MAPK (PD). * p<0.05, n = 3.

Previous studies have suggested that ADAM17 activation is associated with its translocation to the cell surface, which means that only the active form of ADAM17 can be located at the cell surface [11], [48]. In order to elucidate whether the treatment of 50 mg/kg of BPA or NP in young rats induces ADAM17 translocation to the cell surface, as a parameter of its activation, we isolates cells within the seminiferous tubules of rat testes 24 h after treatment. Live cells were incubated with an antibody against the active form of ADAM17 and the labeled cells were evaluated by flow cytometry (for details see Materials and Methods). We found that BPA and NP treatment induced a significant increase in the percentage of cells harboring ADAM17 at their surfaces (Fig. 4). Interestingly, the intra-testicular administration of the ADAM17 inhibitor GW280264X significantly prevented the surface localization of this enzyme in BPA- (a decrease of 65%) but not NP-treated animals (Fig. 4). This difference could be a consequence of the action of BPA and NP on different cell types, or it could be related to the activation of alternative signaling pathways by these compounds. Thus, *in vivo*, BPA and NP induce the translocation of ADAM17 to the cell surface.

Next, we quantified the shedding of TNF-α from rat primary Sertoli cell cultures in the presence of BPA or NP. We chose Sertoli cells as a model system because they present several advantages: 1) They endogenously express ADAM17 [4] and substrates of this enzyme (e.g. TNF-α; 2) For *in vitro* studies, it is possible to isolate a more homogenous cell population than using germ cells; 3) Previous studies have shown that BPA and NP induce apoptosis of Sertoli cells and elicit the activation of signaling events such as the ERK and PTEN/Atk pathway [49], [50]; and 4) It has been shown that MEHP induces shedding of TNF-α from Sertoli cells, suggesting a role for ADAM17 in this process [33].

**Figure 5:**
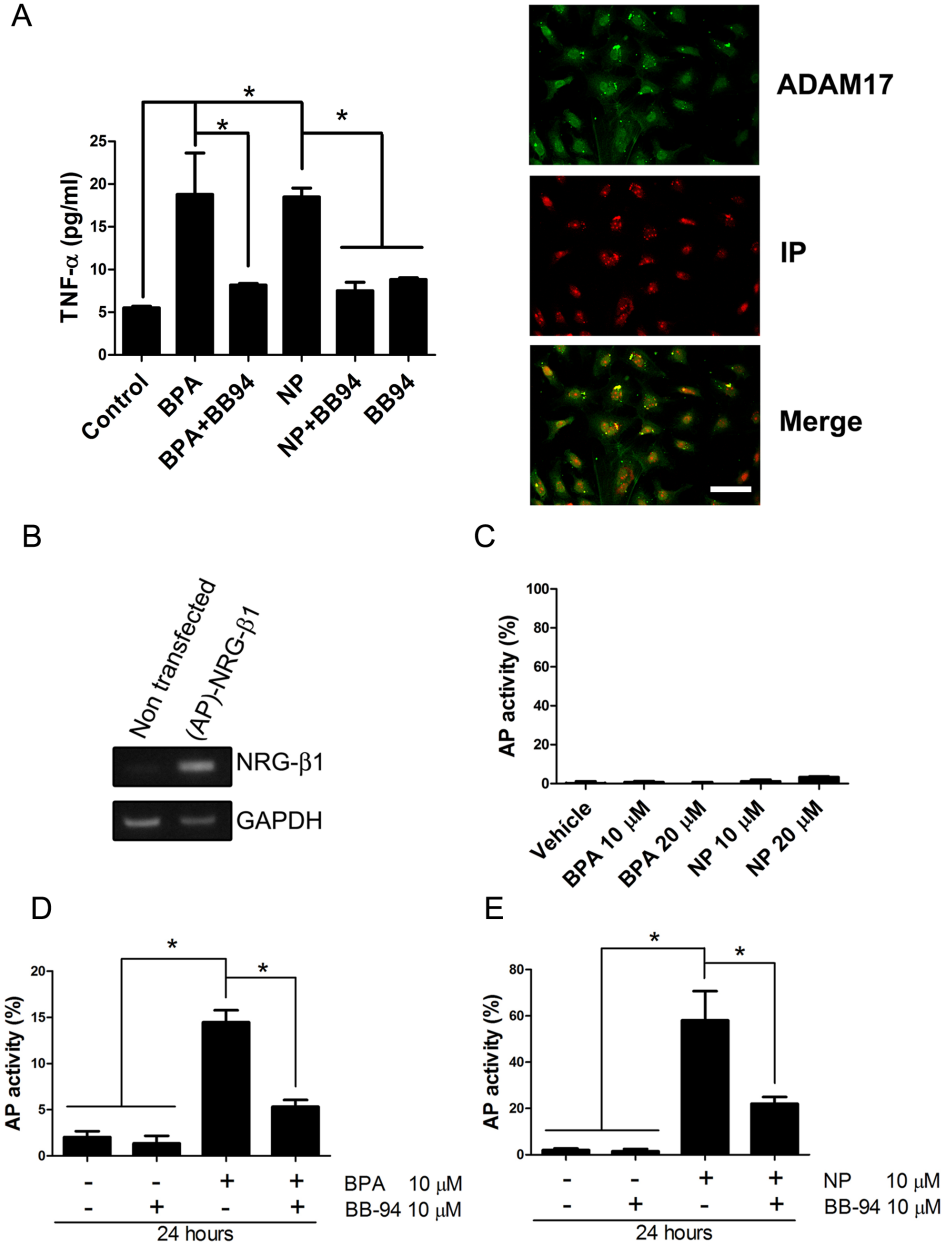
BPA and NP induce shedding of TNF-α and Neuregulin in cultured rat Sertoli cells. A) The amount of TNF-α in the culture medium of primary rats Sertoli cells significantly increases after 6 h of incubation with 10 µM BPA or NP, but remains similar to control levels in the presence of 10 µM BB-94, a general metalloprotease inhibitor. The picture shows immunofluorescence against ADAM17 (green) in rat primary Sertoli cells; cell nuclei are in red stained with Propidium iodide (IP). B) The amplicon of NGR-β1 is detected only in transfected rat primary Sertoli cells with the (AP)-NGR-β1 vector. C) Non-transfected primary Sertoli cells show no AP activity, at any of the tested concentrations. D) BPA and NP (E), induce shedding of AP activity in culture medium after 24 hours of stimulation, which is prevented by BB-94. * p<0.05, n = 3. Bar = 50 µm.

Rat Sertoli cells were grown as mentioned in the Materials and Methods section, and treated with 10 µM of BPA or NP for 6 h; the presence of TNF-α in the culture medium was measured using a commercial kit. The treatment significantly induced TNF-α release, reaching concentrations of 18.79±4.83 pg/mL and 18.50±1.02 pg/mL with BPA and NP, respectively (Fig. 5A). The application of BB-94, a metalloprotease inhibitor, reduced the shedding of TNF-α to values similar to those of controls (8.67±0.19 pg/mL and 7.50±1.00 pg/mL, for BPA and NP, respectively), suggesting the involvement of ADAM17 in the shedding of TNF-α after BPA or NP treatment.

**Figure 6:**
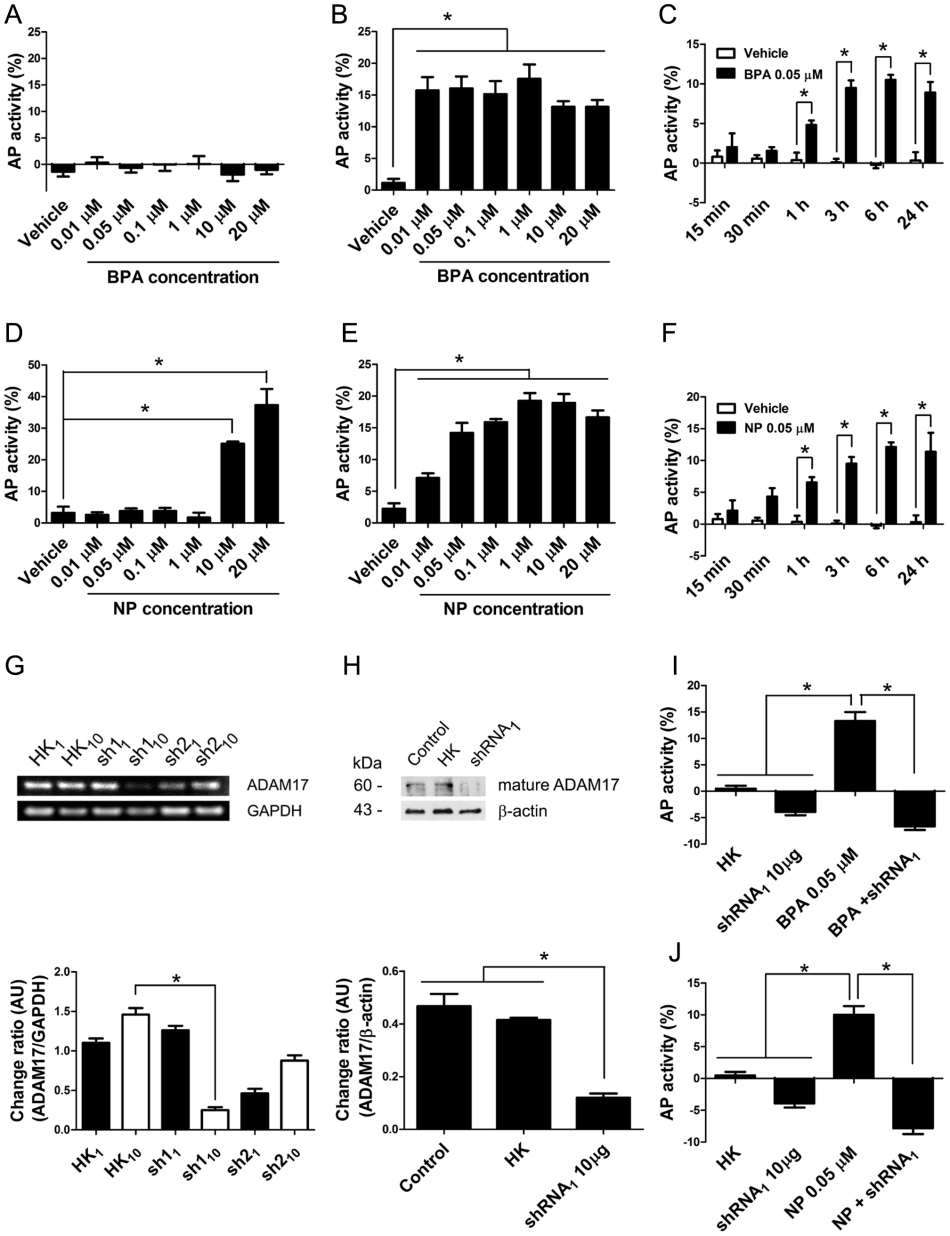
BPA and NP induce the sheddase activity in TM4 cells. A) Non-transfected TM4 cells do not show any detectable shed of AP activity in the culture medium when incubated with BPA. However, NP induces the increase of AP in non-transfected TM4 cells only at concentrations of 10 and 20 µM (D). B, E) The activity of AP significantly increases in the culture medium of transiently transfected TM4 cell with (AP)-NGR-β1 vector, when they are incubated for 24 h in the presence of 0.01–20 µM BPA (B) or NP (E). (C, F) Time course of AP activity release of transiently transfected TM4 cells with (AP)-NGR-β1 using 0.05 µM BPA (C) or NP (F). G) Transfection of TM4 cells with 10 µg of shRNA (clone 1, sh1 10) induces a robust decrease in the levels of mRNA and (H) protein levels of ADAM17. I, J) Silencing of ADAM17 with 10 µg of shRNA1 (shRNA1 10) in transiently transfected TM4 cells with (AP)-NGR-β1 slightly reduces the basal levels of AP shedding and completely prevents the effect of BPA and NP. * p<0.05, n = 3.

Next, we transiently transfected primary Sertoli cells with a construct ((AP)-NGR-β1) expressing the cytoplasmic and transmembrane regions, as well as part of the extracellular domain, of neuregulin-β1 fused to an alkaline phosphatase (AP). This fusion protein has been used in previous works to evaluate the sheddase activity of ADAM17 *in vitro* [51]. In this system, the activity of ADAM17 was studied by measuring the levels of AP in the culture medium of these cells. Cultured Sertoli cells did not express Neuregulin-β1 (NRG-β1), as evaluated by RT-PCR, but a clear amplicon of 325 bp was readily detected only in transfected cells (Fig. 5B). Non-transfected rat primary Sertoli cells did not have AP activity in culture medium, even when using high concentrations of BPA and NP (20 µM) (Fig. 5C). The treatment with 10 µM BPA (Fig. 5D) and NP (Fig. 5E) for 24 h induced a significant increase in the activity of AP, which was prevented by pre-incubation with 10 µM of BB-94, a broad metalloprotease inhibitor (Fig. 5D, E). In order to determine whether these results were restricted to primary rat Sertoli cell cultures, we decided to use the TM4 cell line, which is derived from mouse Sertoli cells [52]. Non-transfected TM4 cells did not express endogenous NGR-β1 (data not shown) and the AP activity was barely detected 24 h after treatment with concentrations ranging from 0.01 to 20 µM BPA or 0.01 to 1 µM NP (Fig. 6A, D). AP activity significantly increased when nontransfected cells were treated with 10 or 20 µM NP suggesting that these cells released the endogenous enzyme at these concentrations (Fig. 6D). TM4 cells that were transiently transfected with (AP)-NGR-β1 plasmid showed a significant increase of AP activity in the culture medium when treated with concentrations ranging from 0.01 to 20 µM NP or BPA for 24 h (Fig. 6B, E). We decided to use a concentration of 0.05 µM of NP and BPA for the following experiments as this concentration was found to significantly increase the AP activity and is within the range of levels detected in human samples [28], [30], [53], [54]. The AP activity started to increase in the culture medium as early as 1 h after treatment with 0.05 µM BPA or NP (Fig. 6C, F). In order to define whether ADAM17 was the enzyme responsible for the increase in AP activity in the culture medium after BPA or NP treatment, we decided to knockdown its expression using specific shRNAs against ADAM17. TM4 cells were transiently transfected with (AP)-NGR-β1 and 1 or 10 µg of shRNAs against ADAM17 (sh1 or sh2 clones), or the empty vector (HK). Results showed that 10 µg of sh1 clone was sufficient to significantly reduce (83%) the mRNA and protein levels (60%) of ADAM17 48 h after transfection (Fig. 6G, H). Since the sh2 clone did not significantly reduce the mRNA levels of ADAM17, we only used the sh1 clone. Finally, transfection with 10 µg of sh1 (sh1_10_) reduced the constitutive shedding of AP activity and after treatment with 0.05 µM NP or BPA (Fig. 6I, F). Therefore, these results indicate that BPA and NP, at concentrations similar to those detected in human samples, induce the activation and shedding of ADAM17 substrates *in vitro*.

### p38 MAPK participates in germ cell apoptosis induced by NP and BPA

**Figure 7:**
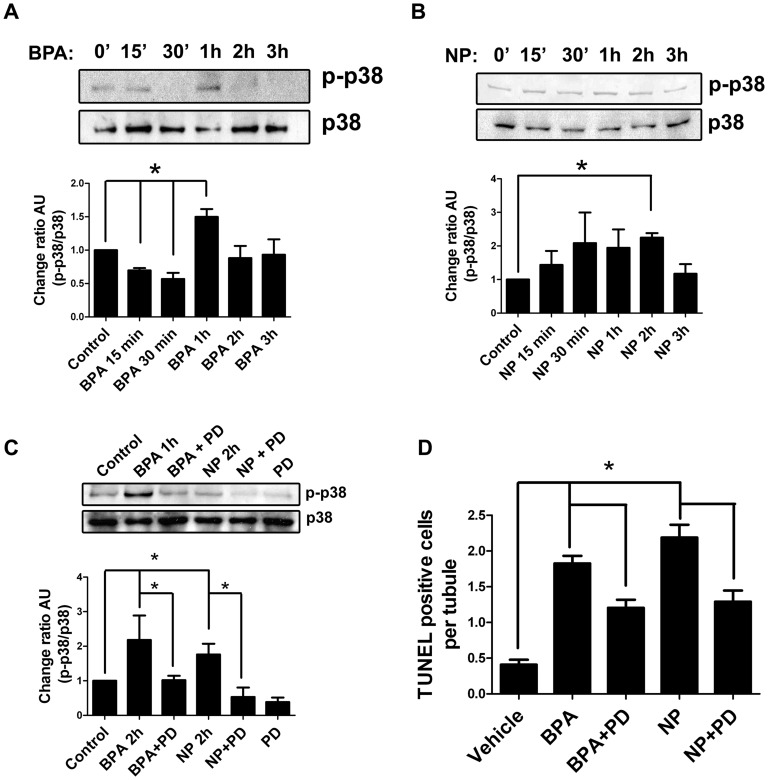
Participation of p38 MAPK in the apoptosis induced by EDC in rat testis. A) Time course of p38 MAPK phosphorylation in 21-day-old rats treated with 50 mg/kg of BPA. B) Time course of p38 MAPK phosphorylation in 21-day-old rats treated with 50 mg/kg of NP. C) Intra-testicular application of 5 µM PD169316 reduces the phosphorylation of p38 MAPK 1 or 2 h after *in vivo* treatment with BPA or NP. D) The pharmacological inhibitor of p38 MAPK (PD169316) prevents the increase of TUNEL-positive cells in testes of 21-day-old rats treated with 50 mg/kg of BPA or NP. * p<0.05, n = 3.

It has been reported that p38 MAPK binds and phosphorylates the cytoplasmic domain of ADAM17 under different conditions [10], [11]. Since p38 MAPK is expressed by germ and Sertoli cells, we decided to evaluate the participation of this kinase in germ cell apoptosis *in vivo* and its relationship with ADAM17 translocation to the cell surface after NP and BPA treatment. For this purpose, 21-day-old rats were injected with 50 mg/kg of BPA or NP and sacrificed at different times (up to 3 hours) to detect the phosphorylated form of p38 MAPK (p-p38). The results showed that 1 h after BPA treatment (Fig. 7A) and 2 h after NP treatment (Fig. 7B), p-p38 was significantly increased. In order to evaluate whether the activation of p38 MAPK was involved in germ cell apoptosis, 5 µM of a pharmacological inhibitor, PD169316, was intra-testicularly injected 1 hour prior to BPA and NP treatment in 21-day-old rats. Levels of p-p38 were similar to those in controls and significantly reduced after treatment with BPA or NP in the presence of PD169316 (Fig. 7C). In addition, PD169316 significantly reduced the percentage of cells harboring ADAM17 at the surface, by 71% and 69% in BPA and NP treatments, respectively (Fig. 4). In the same way, animals treated with PD169316 showed a significant reduction of TUNEL-positive cells from 1.82±0.11 to 1.21±0.11 in the case of BPA, and from 2.19±0.18 to 1.30±0.16 for NP (Fig. 7D); this represents a reduction of BPA- and NP-induced apoptosis by 31% and 39%, respectively, suggesting the participation of p38 MAPK in the apoptosis that is induced by these compounds in the rat testis.

## Discussion

Previous studies have shown that endocrine disruptors induce germ cell apoptosis and reduce male fertility in animal models; however, their effect at the cellular levels seems to be different and the signaling pathways elicited by these compounds are far from elucidated. In this work, we report a novel mechanism by which BPA and NP induce germ cell apoptosis mediated by the activation of p38 MAPK and ADAM17.

Previous work has shown that the chronic administration of BPA and NP to neonatal rats results in histological alterations in the testes, decreased sperm count and morphology and an increase in germ cell apoptosis [31], [32], [44], [55], [56]. In this paper, we extended those results and showed that acute exposure (a single dose of 50 mg/kg) of BPA or NP was sufficient to produce a robust increase in germ cell apoptosis within 24 h, as evaluated by the sub-G1 cell population, active caspase-3 and TUNEL assay (Figs. 1 and 2). Interestingly, we observed that most of the dying germ cells during BPA exposure were cells undergoing meiosis (spermatocytes), whereas those exposed to NP are spermatogonia and few spermatocytes (Fig. 2). These results have been previously observed and suggest that these endocrine disruptors attack different cell types, and may have slightly different mechanisms of action [31], [32]. In addition, these results suggest that chronic exposure to NP might have longer-lasting effects than BPA since it affects spermatogonia rather than spermatocytes. We have shown here that BPA or NP induce apoptosis in germ, but not in Sertoli cells [49], [50], [57]–[60]. However, previous studies have shown that BPA or NP induce apoptosis in Sertoli cells, this discrepancy could be because under *in vitro* conditions Sertoli cells are prone to dead stimuli or that in vivo they have strong survival signals (e.g. extracellular matrix) that protect them from apoptosis. Further studies may be interesting, as they could elucidate the different targets and/or pathways modified by NP and BPA in spermatogonia and spermatocytes.

ADAM17 is a widely expressed enzyme involved in para/juxtacrine signaling in different cell types, which can be activated by different physiological and exogenous stimuli. Our *in vivo* approach showed that an ADAM17 inhibitor, but not an ADAM10 inhibitor, was able to prevent the apoptosis induced by NP and BPA. We also showed that ADAM17 but not ADAM10 inhibitors prevent the increase in TUNEL-positive cells and the cleavage of PARP, which are two *bona fide* apoptosis markers. In order to have some idea of ADAM17 activation *in vivo*, we quantified the percentage of cells from seminiferous tubules expressing this protein at the cell surface. Interestingly, the ADAM17 inhibitor prevented the surface localization of this enzyme after treatment with 50 mg/kg of BPA, but not with NP. This suggests that BPA activates a pathway in which this enzyme participates in its own translocation to the cell surface, similar to that proposed for the cytoplasmic domain phosphorylation of ADAM17 by ERK1/2, which is activated by grown factor receptors, like EGFR, in an ADAM17-dependent manner [61]. Given that BPA and NP induce apoptosis in different cell types (spermatocytes and spermatogonia), another explanation is that the translocation signals of ADAM17 are slightly different between these two cell types. Thus, the difference in translocation to the cell surface in the presence of the ADAM17 inhibitors could represent the response of spermatocytes in the case of BPA, and spermatogonia in the case of NP. Alternatively, it is possible that the translocation of ADAM17 to the cell surface is related to trafficking events rather than its activation. This proposition comes from our data showing that the reduction of the percentage of cells harboring ADAM17 at the surface, which was about 70% when using the ADAM17 (GW280264X) or p38 MAPK (PD169316) inhibitor, was higher than the inhibition of apoptosis, with values of around 50% and 30%, respectively. Overall, these results indicate that BPA and NP induce different responses in the rat testes and induce apoptosis that is dependent on ADAM17 activation.

The *in vitro* results show that NP and BPA induce the shedding of TNF-α from Sertoli cells, and that the knockdown of ADAM17 in TM4 cells completely prevents the shedding of an exogenous substrate (Neuregulin). Therefore, these results suggest that BPA and NP are able to induce ADAM17 activation, and that *in vivo* this enzyme probably sheds one or more specific proteins in Sertoli and germ cells. In this context, it is worth noting that BPA and NP are able to induce ADAM17 activation *in vitro* at concentrations similar to those reported in human blood samples [27], [28], [54], suggesting that these and other similar xenoestrogens could affect physiological processes in humans, different from germ cell apoptosis, such as the release of pro-inflammatory cytokines (e.g. TNF-α) [1], [33], [62], [63]. In addition, these results indicate that, *in vitro*, BPA and NP directly induce the activation of ADAM17 in Sertoli cells from two species (Primary cultures from rat Sertoli cells and the mouse cell line TM4), suggesting that these observation are not restricted to rats and that this effect could probably be extrapolated to humans. In addition, our results suggest that, at least *in vitro*, ADAM17 can be directly activated by BPA and NP and that this effect is not necessarily related to the metabolites of these compounds *in vivo*. Further studies will be need to address the question of whether BPA or NP directly induce the activation of ADAM17 *in vivo*, or if this is accomplished indirectly by promoting the action of other well-known activators of this enzyme, such as Lysophosphatidic Acid (LPA) or Transforming growth factor-alpha (TGF-α) [9], [10], [64].

The sheddase activity of ADAM17 can be regulated at the posttraductional level by different mechanisms, some of which are independent but others which are dependent on the cytoplasmic domain [10], [11], [65], [66]. In addition, it has been proposed that the activation and substrate specificity of ADAM17 requires the presence of ancillary proteins such as iRhom, Annexins or tetraspanin CD9 [31], [67], [68]. In this work, we showed that BPA and NP induce a transient phosphorylation (activation) of p38 MAPK *in vivo* within the first 2 h of treatment. In addition, the inhibitor PD169316 prevents the phosphorylation (activation) of p38 MAPK and the apoptosis induced by BPA and NP, suggesting that activation of this enzyme is important to elicit germ cell demise (Fig. 7A and B). In addition, p38 MAPK has been proposed to be involved in germ cell apoptosis induced by heat stress and hormone deprivation [2], [69], suggesting that different stimuli could induce similar intracellular signaling pathways. BPA and NP could induce p38 MAPK activation by acting as a stress signal rather than as an estrogenic mimic, since p38 MAPK is a well-known marker of cellular stress. The results outlined in this work indicate that activation of p38 MAPK is involved in the increase in cell surface localization of ADAM17 in rat testis cells, since p38 MAPK inhibition by PD169316 significantly reduces the increase in ADAM17 surface levels induced by both BPA and NP. Interestingly, in another study, a reduction of surface ADAM17 was observed, along with cytoplasmic tail phosphorylation, by blocking p38 MAPK in monocytes and lymphocytes [11], suggesting the participation of this kinase in the activation and translocation of ADAM17 to the cell surface. However, the experimental evidence showing that the cytoplasmic domain of ADAM17 is not necessary for its activation suggests that p38 MAPK could act upon ancillary proteins in order to promote its translocation to the cell surface. Alternatively, the translocation of ADAM17 to the cell surface could be related to other process such as cell addition or germ cell apoptosis. However, p38 MAPK could influence apoptosis by other pathways, which could be independent of ADAM17 activation. Thus, the precise link between ADAM17 and p38 MAPK activation remains to be elucidated.

We have previously shown that germ cells undergoing apoptosis lack the extracellular domain of the tyrosine kinase receptor c-kit, suggesting that activation of ADAM17 would shed the extracellular domain of this receptor and this, in turn, will promote apoptosis. We have shown here, at least *in vitro*, that BPA and NP induce the activation and shedding of ADAM17 substrates using Sertoli cells as a model system in two different species. *In vivo*, BPA and NP affect all cell types in the testes, but they elicit different responses. We believe that inside the seminiferous tubules, BPA and NP induce the shedding of different ADAM17 substrates in Sertoli and germ cells. In Sertoli cells, they could act in an autocrine way (e.g. TNF-α) in order to promote the survival of these cells. On the other hand, in germ cells, the activation of ADAM17 would induce shedding of the extracellular domain of c-kit and induce apoptosis. Interestingly, TNF-α is released by germ cells after MEHP-induced Sertoli cell injury and then induces FasL expression, which, in turn, would promote germ cell apoptosis [70]. These previous results fit very well with our model and suggest that the release of TNF-α could act on both autocrine and paracrine pathways in order to promote germ cell apoptosis.
